# Effect of carnosine supplementation on the plasma lipidome in overweight and obese adults: a pilot randomised controlled trial

**DOI:** 10.1038/s41598-017-17577-7

**Published:** 2017-12-12

**Authors:** Estifanos Baye, Jozef Ukropec, Maximilian PJ de Courten, Silvia Vallova, Patrik Krumpolec, Timea Kurdiova, Giancarlo Aldini, Barbara Ukropcova, Barbora de Courten

**Affiliations:** 1 Monash Centre for Health Research and Implementation, School of Public Health and Preventive Medicine, Monash University, Melbourne, Australia; 2 Institute of Experimental Endocrinology, Biomedical Research Centre, Slovak Academy of Sciences, Bratislava, Slovakia; 3 Centre for Chronic Disease, College of Health and Biomedicine, Victoria University, Melbourne, Australia; 4 Institute of Pathological Physiology, Faculty of Medicine, Comenius University, Bratislava, Slovakia; 5 Department of Pharmaceutical Sciences, Università degli Studi di Milano, Milan, Italy

## Abstract

Carnosine has been shown to reduce oxidation and glycation of low density lipoprotein hence improving dyslipidaemia in rodents. The effect of carnosine on human plasma lipidome has thus far not been investigated. We aimed to determine whether carnosine supplementation improves the plasma lipidome in overweight and obese individuals. Lipid analysis was performed by liquid chromatography mass spectrometry in 24 overweight and obese adults: 13 were randomly assigned to 2 g carnosine daily and 11 to placebo, and treated for 12 weeks. Carnosine supplementation maintained trihexosylceramide (0.01 ± 0.19 vs −0.28 ± 0.34 nmol/ml, p = 0.04), phosphatidylcholine (77 ± 167 vs −81 ± 196 nmol/ml, p = 0.01) and free cholesterol (20 ± 80 vs −69 ± 80 nmol/ml, p = 0.006) levels compared to placebo. Trihexosylceramide was inversely related with fasting insulin (r = −0.6, p = 0.002), insulin resistance (r = −0.6, p = 0.003), insulin secretion (r = −0.4, p = 0.05) and serum carnosinase 1 activity (r = −0.3, p = 0.05). Both phosphatidylcholine and free cholesterol did not correlate with any cardiometabolic parameters. Our data suggest that carnosine may have beneficial effects on the plasma lipidome. Future larger clinical trials are needed to confirm this.

## Introduction

Dyslipidaemia is one of the modifiable cardiometabolic risk factors that has been shown to play a significant role in the development of insulin resistance, type 2 diabetes (T2DM) and cardiovascular diseases (CVD)^1–3^. In clinical practice, dyslipidaemia is usually defined as increased total cholesterol and low density lipoprotein (LDL) with decreased high density lipoprotein (HDL) levels^3^. This provides a very narrow snapshot of the dynamic processes involved in lipid metabolism^4^. In addition, these traditional clinical measures are not sufficient to explain the complexity of lipid metabolism which are known to be altered in obese individuals, patients with metabolic syndrome and T2DM, and individuals with high cardiovascular risk^5–7^. Lipidomics approach can provide new insight into the lipid metabolism by offering a chance to examine the lipid classes and species in plasma^4,5^. Lipidomics is focused on the comprehensive identification and quantification of all lipids from serum, plasma, tissue, whole organism or cell^8^. Lipid profiling has shown promising results in the identification of metabolic biomarkers, understanding the mechanisms of lipid metabolism, and diagnosis of various conditions and diseases such as obesity, metabolic syndrome, T2DM and CVD as well as in determining responses to therapeutic interventions^9–12^. Recently, plasma lipid classes and species have been shown to associate with T2DM and CVD^5,13–15^.

Carnosine is an over-the-counter food supplement and it has been shown to prevent and treat several chronic diseases^16^ through its anti-inflammatory, antioxidant, anti-glycation, anti-ischaemic and chelating properties^17–19^. We have previously demonstrated that carnosine supplementation prevented worsening of insulin resistance in non-diabetic overweight and obese individuals, but did not change plasma lipid profiles such as total and HDL cholesterols, and triglycerides^20^. However, in both diabetic and non-diabetic rodents, carnosine has been shown to improve dyslipidaemia, reduce oxidation and glycation of LDL and reduce development of atherosclerosis^21,22^. We have therefore hypothesised that carnosine supplementation may have beneficial effect on the plasma lipidome in overweight and obese individuals which will be related to cardiometabolic parameters.

## Results

### Baseline characteristics

Twenty-four participants (13 in carnosine, 11 placebo group) were included in this analysis. Of these, six of them were females (3 in each group). The overall mean age of the participants’ were 43 ± 7 years with a body mass index of 31 ± 4.5 kg/m^2^. Baseline anthropometric and blood pressure measurements, glucose parameters, lipid profiles, inflammatory markers, urinary carnosine levels, and serum carnosinase activity and content were not different between the groups (Table 1). Dietary fat preference and resting energy expenditure did not differ between the groups. Similarly, levels of lipid classes at baseline were not different in carnosine and placebo groups (Table 2).

**Table 1:** Participant baseline characteristics.

**Parameters**	**Carnosine (n = 13)**	**Placebo (n = 11)**	**P-value**
Sex	F = 3, M = 10	F = 3, M = 8	
Age (years)	42 ± 6	43 ± 9	0.73
Body mass index (kg/m2)	30.4 ± 4.5	32.3 ± 4.6	0.32
Waist-to-hip ratio	0.89 ± 0.06	0.88 ± 0.06	0.74
Systolic BP (mm Hg)	117.1 ± 8.8	125.1 ± 13.3	0.09
Diastolic BP (mm Hg)	76.1 ± 5.5	83.1 ± 9.2	0.06
Fasting glucose (mmol/l)	5.4 ± 0.46	5.3 ± 0.50	0.60
2 h glucose (mmol/l)	6.6 ± 2.0	7.5 ± 2.4	0.35
Fasting insulin (mU/l)	12.4 ± 7.3	14.3 ± 5.4	0.49
2 h insulin (mU/l)	109.5 ± 75.9	127.5 ± 84.6	0.58
HOMA-IR	3.0 ± 2.0	3.2 ± 1.2	0.69
HOMA-B	136.3 ± 55.1	182.1 ± 89.9	0.14
Total cholesterol (mmol/l)	5.4 ± 0.9	5.3 ± 0.8	0.77
High density lipoprotein (mmol/l)	1.2 ± 0.3	1.2 ± 0.2	0.97
Triglycerides (mmol/l)	1.8 ± 1.0	1.5 ± 0.5	0.41
C-reactive protein (mg/l)	3.4 ± 2.9	4.4 ± 4.6	0.50
Adiponectin (µmol/ml)	5.9 ± 3.3	5.8 ± 1.8	0.96
Urinary carnosine levels (nmol/ml)	8.9 ± 6.1	8.0 ± 6.3	0.73
Carnosinase 1 activity (µM)	1.8 ± 0.8	2.1 ± 0.6	0.39
Fat preference score (high fat/low fat score)	0.94 ± 0.13	0.86 ± 0.12	0.12
REE (kcal/kg/day)	32.3 ± 4.6	32.3 ± 6.5	0.99

Means and standard deviations were reported. Independent t-tests were computed to determine the p-value.

BP, blood pressure; HOMA-B, homeostatic model of assessment of insulin secretion; HOMA-IR, homeostatic model of assessment of insulin resistance; REE, resting energy expenditure.

**Table 2:** Effect of carnosine supplementation on the plasma lipidome.

**Lipid class**	**Carnosine group (n = 13)**		**Placebo group (n = 11)**		P*	**P** ^**#**^
	**Baseline**	**Change from baseline**	**Baseline**	**Change from baseline**		
dhCer	0.93 ± 0.22	0.19 ± 0.24	0.99 ± 0.31	0.02 ± 0.27	0.53	0.15
Cer	7.26 ± 1.47	0.06 ± 1.03	6.65 ± 1.24	−0.22 ± 0.63	0.28	0.08
MHC	19.22 ± 4.34	−0.78 ± 3.54	16.28 ± 3.57	−0.86 ± 1.09	0.08	0.16
DHC	9.29 ± 1.82	−0.66 ± 1.13	8.60 ± 1.53	−0.95 ± 1.45	0.33	0.21
THC	**1.78 ± 0.43**	**0.01 ± 0.19**	**1.98 ± 0.45**	**−0.28 ± 0.34**	**0.26**	**0.04**
GM3	5.34 ± 1.27	−0.09 ± 0.55	4.99 ± 1.04	−0.38 ± 0.56	0.48	0.20
SM	421.38 ± 73.29	−9.74 ± 36.72	425.35 ± 98.92	−31.23 ± 54.22	0.91	0.10
PC	**1821.96 ± 264.12**	**77.28 ± 16.70**	**1801.78 ± 282.53**	**−81.27 ± 196.02**	**0.85**	**0.01**
PC(O)	82.05 ± 12.17	1.27 ± 14.86	90.43 ± 15.54	−3.59 ± 11.64	0.15	0.84
PC(P)	41.35 ± 5.98	0.49 ± 5.81	45.35 ± 11.45	−2.26 ± 8.07	0.28	0.67
LPC	118.67 ± 14.52	−5.15 ± 19.41	105.99 ± 24.54	−10.20 ± 14.21	0.13	0.14
LPC(O)	1.71 ± 0.11	−0.14 ± 0.25	1.69 ± 0.20	−0.01 ± 0.13	0.85	0.68
PE	32.63 ± 9.51	−0.046 ± 5.91	31.51 ± 11.16	−0.10 ± 7.29	0.79	0.81
PE(O)	2.69 ± 1.00	0.14 ± 1.13	2.92 ± 1.00	−0.08 ± 0.97	0.59	0.82
PE(P)	29.20 ± 5.41	2.79 ± 8.09	31.66 ± 5.41	0.19 ± 7.33	0.27	0.76
LPE	7.18 ± 1.47	−0.16 ± 1.73	6.41 ± 1.26	−0.35 ± 0.97	0.18	0.17
PI	44.22 ± 12.45	−1.71 ± 6.40	39.95 ± 8.58	−2.06 ± 6.43	0.34	0.27
LPI	0.74 ± 0.17	0.098 ± 0.19	0.73 ± 0.17	0.06 ± 0.13	0.88	0.49
PS	9.07 ± 3.09	0.05 ± 2.56	10.39 ± 4.53	−1.77 ± 5.89	0.40	0.55
PG	0.22 ± 0.13	−0.01 ± 0.07	0.18 ± 0.008	−0.01 ± 0.07	0.39	0.49
CE	1345.49 ± 251.06	5.49 ± 21.05	1254.97 ± 206.26	−22.47 ± 174.38	0.35	0.58
COH	**896.96 ± 131.96**	**20.05 ± 79.62**	**933.82 ± 166.13**	**−68.56 ± 79.71**	**0.55**	**0.006**
DG	40.86 ± 24.67	1.03 ± 15.39	35.88 ± 16.95	−0.04 ± 20.03	0.57	0.78
TG	347.22 ± 175.29	19.40 ± 129.72	289.03 ± 93.68	−4.31 ± 9.13	0.33	0.58

All values are in nmol/ml. Means and standard deviations were reported.

*Independent t-tests were conducted to compare the baseline differences between the groups.

^#^Analysis of covariance were performed to determine between-group treatment differences.

dhCer, dihydroceramide; Cer, ceramide; MHC, monohexosylceramide; DHC, dihexosylceramide; THC, trihexosylcermide; GM3, GM3 ganglioside; SM, sphingomyelin; PC, phosphatidylcholine; PC(O), alkylphosphatidylcholine; PC(P), alkenylphosphatidylcholine; PC, lysophosphatidylcholine; LPC(O), lysoalkylphosphatidylcholine; PE, phosphatidylethanolamine; PE(O), alkylphosphatidylethanolamine; PE(P), alkenylphosphatidylethanolamine; LPE, lysophosphatidylethanolamine; PI, phosphatidylinositol; LPI, lysophosphatidylinositol; PS, phosphatidylserine; PG, phosphatidylglycerol; CE, cholesterol ester; COH, free cholesterol; DG, diacylglycerol; TG, triacylglycerol.

### Effect of carnosine supplementation on the plasma lipidome

Carnosine supplementation maintained the levels of trihexosylceramide (THC) (mean change from baseline ± standard deviation: 0.01 ± 0.19 versus −0.28 ± 0.34 nmol/ml, p = 0.04) and phosphatidylcholine (PC) (77 ± 167 versus −81 ± 196 nmol/ml, p = 0.01) lipid classes compared to placebo. Free cholesterol (COH) was also preserved in the carnosine group than placebo (20 ± 80 versus −69 ± 80 nmol/ml, p = 0.006). None of the other lipid classes showed changes between the groups (Table 2).

### Relationship between lipid classes and cardiometabolic parameters

Change in THC was inversely related with change in fasting insulin levels (r = −0.6, p = 0.002), HOMA-IR (r = −0.6, p = 0.003), HOMA-B (r = −0.4, p = 0.05) and serum carnosinase 1 activity (r = −0.5, p = 0.01). These associations except for HOMA-B were significant after adjustment for age, sex, and change in BMI (all p < 0.03). THC did not correlate with other cardiometabolic parameters and carnosine measurements (all p > 0.2). PC was positively correlated with BMI (r = 0.4, p = 0.04), however this relationship was not significant after adjusting either for age or sex (all p > 0.05). Both PC and COH did not associate with anthropometric measures, metabolic or cardiovascular parameters, and urinary carnosine levels as well as serum carnosinase 1 activity/content (all p > 0.1). THC, PC and COH were not related either with dietary fat preference or resting energy expenditure (all p > 0.05).

## Discussion

We measured the effect of carnosine supplementation on the plasma lipidome in non-diabetic overweight and obese adults from a randomised double-blind placebo controlled pilot trial. We have demonstrated that supplementation with 2 g carnosine daily for 12 weeks resulted in changes in plasma lipidome which were associated with improved insulin sensitivity and secretion as well as serum carnosinase 1 activity.

We have demonestrated that THC levels were maintained after carnosine supplementation compared to placebo, which showed a relative increase in the carnosine group. This finding is consistent with the recent data that showed higher levels of THC after lifestyle intervention (diet and exercise) in patients with metabolic syndrome compared to dietary intervention only and no intervention^23^. Treatment with RVX-208, first-in-class BET inhibitor with apolipoprotein A-I inducing effects, has also been shown to increase THC levels in prediabetes males compared to placebo^24^. THC constitutes the main components of cell membranes and has been suggested to have beneficial roles in signal transmission, cell adhesion, growth factor regulation and protein transport^25^. These mechanisms have been shown to play a role in the development of insulin resistance and T2DM^26^. Meikle and colleagues have reported that THC was inversely associated with obesity^27^, plasma glucose level and decreased with prediabetes and T2DM^14^. Lower levels of THC was also observed in people with metabolic syndrome^23^. Similarly, we have found that THC was inversely associated with fasting insulin, insulin resistance, and insulin secretion. We showed, however, no association with other glycaemic measures (fasting glucose, 2 h glucose and insulin levels), anthropometric measures and inflammatory markers. This is likely because our study population was overweight and obese but did not have with other features of metabolic syndrome. Importantly, THC was inversely associated with serum carnosinase 1 activity. Low serum carnosinase 1 activity increases circulating carnosine in humans^28^. We did not, however, find any association between THC and urinary carnosine levels. Nonetheless, we have previously shown that supplementation with carnosine increased the level of carnosine in urine and prevented worsening of insulin sensitivity in non-diabetic overweight and obese adults^20^. Therefore, the observed effect of carnosine on THC levels may suggest its promising effect in normalising the plasma lipid profile in high risk groups which may have a role in preventing the development of insulin resistance and T2DM.

We report that supplementation with carnosine, as compared to placebo, improved plasma PC levels in overweight and obese, otherwise healthy individuals. In line with this, a study that involved older patients with established CVD showed an increased plasma PC levels after treatment with rosuvastatin^29^. A single session of acute exercise has also tended to increase muscle PC levels in patients with T2DM^30^. PC is a major constituent of the plasma membrane and a key element of very-low-density lipoproteins. It serves as a precursor of signalling molecules^31^ and plays a role in exporting triglycerides to the organs^32^. A deficiency of PC in the secretory pathway or in the nascent particle may limit the secretion of very-low-density lipoproteins and leads to the accumulation of hepatic triglycerides^32^. Low cellular PC levels have been shown to activate sterol regulatory element-binding protein-1 (SREBP1), a transcription factor involved in glucose metabolism, thereby contribute to the development of obesity, insulin resistance and fatty liver disease^33^. In support of this, previous reports demonstrated that PC was inversely related with insulin resistance^30,34^, T2DM^35^ and hepatic steatosis^31^. In our study, PC was not related with any cardiometabolic parameters. This could be due to the small sample size of the study or that our participants were all non-diabetic. Most importantly, carnosine supplementation has been shown to diminish the activity of SREBPs, reduce hepatic triglycerides, and improve insulin sensitivity in mice^19^. The beneficial effect of carnosine on PC levels may therefore contribute to the prevention of T2DM and CVD via regulation of SREBP-1 activity.

We find that carnosine supplementation preserved plasma COH levels compared to placebo. A study in people with dyslipidaemia however showed a reduction in COH levels after treatment with simvastatin, as compared to placebo^36^. Another study also reported lower COH levels in patients with T2DM on statins than in those who were not^37^. Whilst these studies demonstrate the efficacy of statins in reducing COH levels in individuals with pre-existing altered lipid metabolism, supplementation with carnosine preserved COH levels in obese and overweight people with normal lipid profiles. Related to this, both high and low levels of COH have been shown to increase the risk of T2DM and CVD^38–40^. COH also has both anti- and pro-inflammatory roles^41,42^. These indicate that COH has U shape relationship with risk of T2DM and CVD^40^. COH is an essential component of cell membranes and is present in tissues or plasma lipoprotein. COH levels appear to be a physiological constant that may be of considerable value in cholesterol metabolism^43^. Alterations in cholesterol metabolism could influence COH accumulation^40^. Obesity, insulin resistance and T2DM are associated with changes in cholesterol metabolism^44^. Elevated COH was observed in obesity^34^ and prediabetes^14^. In addition, COH was positively correlated with total and LDL-cholesterols in overweight and obese children^45^. Although we did not find any association between COH levels and cardiometabolic parameters, which could be due to that our participants were all healthy, the observed role of carnosine on COH levels may help to delay the development of T2DM and CVD through improving cholesterol metabolism. Our findings, however, should be confirmed in larger sample sizes.

### Strengths and Limitations of the study

The study participants underwent a comprehensive metabolic profiling and were not different at baseline between the groups. Rigorous randomisation process was conducted in addition to blinding of the investigators, study personnel and study participants. We have analysed 24 lipid classes from 324 lipid species. Small sample size of the study could be considered as the main limitations of the study. Due to this, p-values for treatment difference between groups were not corrected for multiple comparisons, and the effect of carnosine on each lipid species were not analysed. Further studies using larger sample sizes are needed to confirm these preliminary findings, and determine the effect of carnosine supplementation on plasma lipid species as well.

### Conclusions and Implications for clinical practice

In this exploratory analysis, we have demonstrated for the first time an effect of supplementation with carnosine on THC, PC and COH levels which are likely to have biological roles in the development of insulin resistance, T2DM and CVD. Although lifestyle interventions are effective strategies for prevention and treatment of T2DM and CVD, they are costly and difficult to implement at the population level. Carnosine, a safe and cheap over-the-counter food supplement, may therefore be an effective strategy to prevent or delay the development of insulin resistance and T2DM through improving the plasma lipid profile & metabolism. Future studies in larger sample sizes however are required to confirm these findings as well as to determine the putative effect of carnosine on each plasma lipid species.

## Methods and Materials

### Study design and population

Details of the trial protocol as well as primary outcomes have been published previously^20^. In brief, a randomised, double-blind, placebo-controlled trial was conducted in 30 healthy overweight and obese individuals at the Institue of Experimental Endocrinology, Slovak Academy of Sciences, Slovakia. Eligible participants underwent a rigorous medical screening and they were asked to refrain from strenuous exercise and caffeine for 3 days prior to metabolic testing. All participants were non-diabetic as indicated by a 75 g oral glucose tolerance test (OGTT), non-smokers, and healthy according to a physical examination and routine blood analyses. Participants were randomly assigned either to receive 2 g  carnosine or identical placebo (2 g sucrose) daily (administered orally in two equivalent doses) for 12 weeks. No participant had signs of infection or took any medication or food supplements at the time of the study. Blood and urine collections were performed after a 12 h overnight fast and 12 h after carnosine ingestion. Participants were asked to refrain from substantial lifestyle changes during the course of the study. As such, participants with weight change ≥5 kg in the study period would be excluded from the study. We have excluded 3 participants (1 carnosine, 2 in placebo) for non-compliance with the protocol and 3 (2 carnosine, 1 placebo) participants had missing plasma samples.

All volunteers were recruited from the community and written informed consent was taken prior to study entry. The protocol was approved by the Ethics Committee of the University Hospital Bratislava, Comenius University, Bratislava, Slovakia, and it conforms to the Ethical Declaration of Helsinki.

### Anthropometric and blood pressure measurements

Body weight and height were measured and used to calculate body mass index (BMI). Blood pressure was measured in a sitting position after 30 minutes of rest using a Dinamap Compact (Johnson & Johnson Inc., UK). It was recorded three times, separated by 5 minutes, and the mean value was reported.

### Assessment of dietary preference and resting energy expenditure

Food preference questionnaire was used to determine the participants’ dietary preference for high-fat/low-fat foods based on 72 different food items. Resting energy expenditure (REE) was measured after an overnight fast by indirect calorimetry (Geratherm, Germany).

### Metabolic studies

OGTTs were performed after a 12 h overnight fast and blood samples were collected in every 30 minutes for two hours to determine glucose tolerance status (American Diabetes Association criteria, 2006). Serum glucose was analysed using glucosehexokinase 3 kit (Siemens Health Care Diagnostics, Germany) and insulin was determined with immunoradiometric assays (Immunotech, France). Insulin sensitivity and insulin secretion was calculated using homeostatic model assessment of insulin resistance (HOMA-IR) and insulin secretion (HOMA-B) respectively. Total cholesterol, HDL, and triglycerides were measured using diagnostic kits from Roche (Germany). Hypersensitive C - reactive protein (hsCRP) and adiponectin were analysed by an immunoturbidimetric method (Randox, UK).

### Measurement of carnosine levels and carnosinase activity

Urinary carnosine was measured using an internal standard and a triple quadrupole (TSQ QUANTUM ULTRA, Thermo Scientific, Italy), as previously described^20,46^. Serum carnosinase activity was quantified by fluorometric determination of liberated histidine after carnosine addition, and carnosinase content in serum was determined by a sandwich enzyme-linked immunosorbent assay (ELISA) developed by Adelmann^47^, and as previously described^20,48^.

### Lipidomic analysis

Lipid analysis was performed by liquid chromatography electrospray ionisation-tandem mass spectrometry using an Agilent 1200 liquid chromatography system combined with an Applied Biosystems API 4000 Q/TRAP mass spectrometer with a turboionspray source (350 °C) and Analyst 1.5 data system at Baker IDI Heart and Diabetes Institute, Australia. The detailed methods of analysis has been previously described elsewhere^27^. Briefly, plasma samples (10 mL) were randomised (maintaining paired samples at baseline and after intervention), then extracted in a single-phase extraction with 20 volumes of CHCl3:MeOH (2:1) and 10 mL of an internal standard mix that contained between 50 and 1000 pmol each of 24 non physiologic and stable isotope-labelled lipid standards. The concentration of each lipid species was calculated by relating the peak area of each individual lipid species to the peak area of the corresponding internal standard. The integration of lipid chromatographic peaks was carried out by MassHunter Workstation Software (Agilent Technologies, USA). The concentration of individual lipids was calculated by relating the peak area of each lipid species to the peak area of the corresponding internal standard. A total of 324 lipid species in 24 lipid classes were analysed by multiple reaction monitoring experiments. The totals for each lipid class were calculated by adding the concentrations of individual lipid species within the same class^27^. Whole plasma was analysed in triplicate and the mean values of each triplicate subsequently used for statistical analysis. Assay performance was monitored by calculating the coefficient of variation (percent) of the quality control plasma samples across the entire analytical run. The median coefficient of variation of the internal standard areas and plasma quality control samples were 10% and 7%, respectively, which indicate that the measurement had good precision (≤15%).

### Statistical analysis

The sample size calculation was computed based on the primary outcome, insulin sensitivity, and has been reported elsewhere^20^. Data analyses were performed using Stata V.14 (StatCorp LP, USA). Means and standard deviations were reported, unless otherwise stated. Appropriate data transformations were performed when needed. Independent student t-tests were conducted to compare the baseline participant characteristics between carnosine and placebo groups. Treatment differences between the groups (between-group differences) were compared using analysis of covariance with the use of baseline values as covariates. Pearson correlations were used to determine the relationship between the lipid classes and cardiometabolic parameters. Linear regressions were performed to confirm the presence of the associations between lipid classes and cardiometabolic parameters after taking into account age, sex and BMI differences.

### Data availability

The datasets generated during and/or analysed during the current study are available from the corresponding author on reasonable request.

## References

[1] Zhou X, Zhang W, Liu X, Zhang W, Li Y (2015). Interrelationship between diabetes and periodontitis: role of hyperlipidemia. Arch Oral Biol.

[2] Vergani, C. & Lucchi, T. Plasma HDL cholesterol and risk of myocardial infarction. *Lance*t **38**0, 1990, author reply 1991, 10.1016/s0140-6736(12)62148-5 (2012). 10.1016/S0140-6736(12)62148-5 23217859

[3] Meikle PJ, Christopher MJ (2011). Lipidomics is providing new insight into the metabolic syndrome and its sequelae. Curr Opin Lipidol.

[4] Meikle PJ, Wong G, Barlow CK, Kingwell BA (2014). Lipidomics: potential role in risk prediction and therapeutic monitoring for diabetes and cardiovascular disease. Pharmacol Ther.

[5] Stegemann C (2014). Lipidomics profiling and risk of cardiovascular disease in the prospective population-based Bruneck study. Circulation.

[6] Haus JM (2009). Plasma ceramides are elevated in obese subjects with type 2 diabetes and correlate with the severity of insulin resistance. Diabetes.

[7] Stefan N, Schick F, Haring HU (2014). Ectopic fat in insulin resistance, dyslipidemia, and cardiometabolic disease. N Engl J Med.

[8] Spener F, Lagarde M, Géloên A, Record M (2003). Editorial: What is lipidomics?. European Journal of Lipid Science and Technology.

[9] Zhao YY, Miao H, Cheng XL, Wei F (2015). Lipidomics: Novel insight into the biochemical mechanism of lipid metabolism and dysregulation-associated disease. Chem Biol Interact.

[10] Hu C (2011). Application of plasma lipidomics in studying the response of patients with essential hypertension to antihypertensive drug therapy. Mol Biosyst.

[11] Kien CL (2013). A lipidomics analysis of the relationship between dietary fatty acid composition and insulin sensitivity in young adults. Diabetes.

[12] Kwan HY (2013). Lipidomics identification of metabolic biomarkers in chemically induced hypertriglyceridemic mice. J Proteome Res.

[13] Greig FH, Kennedy S, Spickett CM (2012). Physiological effects of oxidized phospholipids and their cellular signaling mechanisms in inflammation. Free Radic Biol Med.

[14] Meikle PJ (2013). Plasma lipid profiling shows similar associations with prediabetes and type 2 diabetes. PLoS One.

[15] Meikle PJ (2011). Plasma lipidomic analysis of stable and unstable coronary artery disease. Arterioscler Thromb Vasc Biol.

[16] Baye E (2016). Physiological and therapeutic effects of carnosine on cardiometabolic risk and disease. Amino Acids.

[17] Aldini G (2011). The carbonyl scavenger carnosine ameliorates dyslipidaemia and renal function in Zucker obese rats. J Cell Mol Med.

[18] Sauerhofer S (2007). L-carnosine, a substrate of carnosinase-1, influences glucose metabolism. Diabetes.

[19] Mong MC, Chao CY, Yin MC (2011). Histidine and carnosine alleviated hepatic steatosis in mice consumed high saturated fat diet. Eur J Pharmacol.

[20] de Courten B (2016). Effects of carnosine supplementation on glucose metabolism: Pilot clinical trial. Obesity (Silver Spring).

[21] Lee YT, Hsu CC, Lin MH, Liu KS, Yin MC (2005). Histidine and carnosine delay diabetic deterioration in mice and protect human low density lipoprotein against oxidation and glycation. Eur J Pharmacol.

[22] Rashid I, van Reyk DM, Davies MJ (2007). Carnosine and its constituents inhibit glycation of low-density lipoproteins that promotes foam cell formation *in vitro*. FEBS Lett.

[23] Khan A (2015). Effect of weight loss and exercise on the high-density lipoprotein (HDL) lipidome in individuals with metabolic syndrome (METS). Atherosclerosis.

[24] Siebel AL (2016). Effects of the BET-inhibitor, RVX-208 on the HDL lipidome and glucose metabolism in individuals with prediabetes: A randomized controlled trial. Metabolism.

[25] Xu YH, Barnes S, Sun Y, Grabowski GA (2010). Multi-system disorders of glycosphingolipid and ganglioside metabolism. J Lipid Res.

[26] Larsen PJ, Tennagels N (2014). On ceramides, other sphingolipids and impaired glucose homeostasis. Mol Metab.

[27] Weir JM (2013). Plasma lipid profiling in a large population-based cohort. J Lipid Res.

[28] Everaert I (2012). Low plasma carnosinase activity promotes carnosinemia after carnosine ingestion in humans. Am J Physiol Renal Physiol.

[29] Bergheanu SC (2008). Lipidomic approach to evaluate rosuvastatin and atorvastatin at various dosages: investigating differential effects among statins. Curr Med Res Opin.

[30] Newsom SA (2016). Skeletal muscle phosphatidylcholine and phosphatidylethanolamine are related to insulin sensitivity and respond to acute exercise in humans. J Appl Physiol (1985).

[31] Vance JE, Vance DE (2004). Phospholipid biosynthesis in mammalian cells. Biochem Cell Biol.

[32] Noga AA, Vance DE (2003). A gender-specific role for phosphatidylethanolamine N-methyltransferase-derived phosphatidylcholine in the regulation of plasma high density and very low density lipoproteins in mice. J Biol Chem.

[33] Walker AK (2011). A conserved SREBP-1/phosphatidylcholine feedback circuit regulates lipogenesis in metazoans. Cell.

[34] Graessler J (2009). Top-down lipidomics reveals ether lipid deficiency in blood plasma of hypertensive patients. PLoS One.

[35] Rhee EP (2011). Lipid profiling identifies a triacylglycerol signature of insulin resistance and improves diabetes prediction in humans. J Clin Invest.

[36] Chen F (2011). The effects of simvastatin treatment on plasma lipid-related biomarkers in men with dyslipidaemia. Biomarkers.

[37] Feitosa-Filho GS, Seydell Tde M, Feitosa AC, Maranhao RC, Ramires JA (2009). Lipid transfer to HDL in type-2 diabetic patients: associations with microalbuminuria, statin, and insulin. Arq Bras Cardiol.

[38] Kockx M (2012). Cholesterol accumulation inhibits ER to Golgi transport and protein secretion: studies of apolipoprotein E and VSVGt. Biochem J.

[39] Tabas I (2010). The role of endoplasmic reticulum stress in the progression of atherosclerosis. Circ Res.

[40] Xu X, Zhang A, Li N, Li PL, Zhang F (2015). Concentration-Dependent Diversifcation Effects of Free Cholesterol Loading on Macrophage Viability and Polarization. Cell Physiol Biochem.

[41] Yao PM, Tabas I (2001). Free cholesterol loading of macrophages is associated with widespread mitochondrial dysfunction and activation of the mitochondrial apoptosis pathway. J Biol Chem.

[42] Peled M, Fisher EA (2014). Dynamic Aspects of Macrophage Polarization during Atherosclerosis Progression and Regression. Front Immunol.

[43] Sperry M (1936). The relationship between total and free cholesterol in human blood serum. J. Biol. Chem.

[44] Miettinen TA, Gylling H (2000). Cholesterol absorption efficiency and sterol metabolism in obesity. Atherosclerosis.

[45] Son HH (2015). Serum sterol profiling reveals increased cholesterol biosynthesis in childhood obesity. J Steroid Biochem Mol Biol.

[46] Pfister F (2011). Oral carnosine supplementation prevents vascular damage in experimental diabetic retinopathy. Cell Physiol Biochem.

[47] Adelmann K (2012). Different conformational forms of serum carnosinase detected by a newly developed sandwich ELISA for the measurements of carnosinase concentrations. Amino Acids.

[48] Stegen S (2015). Muscle histidine-containing dipeptides are elevated by glucose intolerance in both rodents and men. PLoS One.

