# Author Correction: Effect of carnosine supplementation on the plasma lipidome in overweight and obese adults: a pilot randomised controlled trial

**DOI:** 10.1038/s41598-020-61335-1

**Published:** 2020-03-04

**Authors:** Estifanos Baye, Jozef Ukropec, Maximilian P. J. de Courten, Silvia Vallova, Patrik Krumpolec, Timea Kurdiova, Giancarlo Aldini, Barbara Ukropcova, Barbora de Courten

**Affiliations:** 1 Monash Centre for Health Research and Implementation, School of Public Health and Preventive Medicine, Monash University, Melbourne, Australia; 2 Institute of Experimental Endocrinology, Biomedical Research Centre, Slovak Academy of Sciences, Bratislava, Slovakia; 3 Centre for Chronic Disease, College of Health and Biomedicine, Victoria University, Melbourne, Australia; 4 Institute of Pathological Physiology, Faculty of Medicine, Comenius University, Bratislava, Slovakia; 5 Department of Pharmaceutical Sciences, Università degli Studi di Milano, Milan, Italy

Correction to: *Scientific Reports* 10.1038/s41598-017-17577-7, published online 12 December 2017

This Article contains an error in the Data Availability section.

“The datasets generated during and/or analysed during the current study are available from the corresponding author on reasonable request.”

should read:

“Summary data generated during and/or analysed during the current study are available from the corresponding author on reasonable request, but restrictions apply to the availability of the raw data, which is owned by Monash Health, as it details personal patient information.”

